# Antitumor and Antiangiogenic Activities of Curcumin in Cervical Cancer Xenografts in Nude Mice

**DOI:** 10.1155/2014/817972

**Published:** 2014-04-16

**Authors:** Pornphrom Yoysungnoen-Chintana, Parvapan Bhattarakosol, Suthiluk Patumraj

**Affiliations:** ^1^Division of Physiology, Department of Preclinical Science, Faculty of Medicine, Thammasat University, Rangsit Campus, Klong Luang, Pathum Thani 12120, Thailand; ^2^Department of Microbiology, Faculty of Medicine, Chulalongkorn University, Bangkok 10330, Thailand; ^3^Department of Physiology, Faculty of Medicine, Chulalongkorn University, Bangkok 10330, Thailand

## Abstract

To evaluate the effects of curcumin (CUR) on tumor progression and angiogenesis in cervical cancer- (CaSki-) implanted nude mice and on the angiogenic biomarkers: vascular endothelial growth factor (VEGF), cyclooxygenase-2 (COX-2), and epidermal growth factor receptor (EGFR). CaSki cells were subcutaneously injected in nude mice to establish subcutaneous tumors. One month after injection, mice were orally administered vehicle or 500, 1,000, and 1,500 mg/kg of CUR daily × 30 consecutive days. Tumor volume was measured every 3-4 days. At the end of the study, tumor microvasculature was observed under confocal microscope, and immunohistochemical analyses were performed to detect CD31, VEGF, COX-2, and EGFR. CUR at the doses of 1,000 and 1,500 mg/kg showed significant tumor growth retardation (21.03% and 35.57%) versus CaSki + vehicle group. The microvascular density (MVD) in CaSki + vehicle group was significantly increased versus Control + vehicle group and significantly reduced by CUR (1,000 and 1,500 mg/kg). VEGF, COX-2, and EGFR expressions were upregulated in CaSki + vehicle group and attenuated significantly by CUR (1,000 and 1,500 mg/kg). In conclusion, high dose CUR inhibited tumor growth and angiogenesis in CaSki-implanted mice probably mediated by the downregulation of VEGF, COX-2 and EGFR. CUR may have a role in treating human cervical cancer and should be explored further.

## 1. Introduction

Cervical cancer is the second most common cancer in women worldwide and is the most frequent cancer in many developing countries [1]. Cervical tumors are often highly vascular and bleed spontaneously. The addition of antiangiogenic agents could be important in the treatment of human cervical cancer. Antiangiogenic treatment strategies offer a number of compelling advantages over conventional cytotoxic cancer therapies because endothelial cells are not transformed and drug resistance is not induced. In this regard, discovery of nontoxic antiangiogenic phytochemicals could have greater practical significance compared to nonselective cytotoxic therapies to control the tumor growth and metastasis by targeting angiogenesis.

A number of biological molecules modulate angiogenesis in cervical cancer such as epidermal growth factor receptor (EGFR), cyclooxygenase-2 (COX-2), and vascular endothelial growth factor (VEGF). EGFR is a member of the ErbB family, the tyrosine kinase receptors with growth promoting effects, including a recognized angiogenic potential [2]. Activation of EGFR results in activation of MEK-extracellular signal-regulated kinase1/2 (ERK1/2) and phosphatidylinositol 3-kinase (PI3K-) Akt pathways [3]. These two pathways regulate VEGF expression through changes in VEGF transcriptional activity which is a major mediator of tumor angiogenesis. Recently, Balan et al. reported that EGFR was overexpressed in cervical biopsies of cervical cancer patients [4]. The number of biopsies with intense immunoexpression of EGFR increased with the severity of the cytological abnormality. Thus, EGFR seems to have an important role in tumor angiogenesis and the prognostic of advanced cervical cancer.

The VEGF, a major mediator of tumor angiogenesis, promotes mobilization of endothelial progenitor cells, cell proliferation, migration, survival, and vascular permeability [5]. VEGF was found to be overexpressed in cervical cancer and associated with a poor prognosis [6, 7]. The COX-2 has a role in the onset and progression of malignancies, including the cervical carcinoma, and is also considered as a marker of tumor aggressiveness. It can potentially predispose to cervical cancer by several mechanisms. An increased expression of COX-2 has been reported to inhibit apoptosis, suppress immune function, promote angiogenesis, and enhance the invasiveness of malignant cells [8]. Kulkarni et al. found that EGF and TGF-*α*, ligands of EGFR, markedly induced COX-2 in a cervical carcinoma cell line, suggesting that deregulated signaling through EGFR is likely to account, at least in part, for increased expression of COX-2 [9]. Later, Oh et al. reported that COX-2-prostaglandin E2 (PGE2) pathway is also implicated in VEGF expression by HPV 16 E5 [10]. Therefore, EGFR-COX-2-PGE2 pathway also plays an important role in tumor growth and angiogenesis in cervical cancer.

Curcumin (CUR), a phenolic compound extracted from* Curcuma longa *L., is well known as a chemopreventive agent. Many recent studies have demonstrated that CUR modulates angiogenesis, proliferation, invasion, and tumor progress in various types of cancer [8, 11–13]. However, the effect of CUR on tumor angiogenesis, especially, using cervical cancer- (CaSki-) implanted nude mice model has not yet been reported. Therefore, the present study was designed to determine the effects of CUR on angiogenesis and tumor progression in cervical cancer- (CaSki-) implanted nude mice and to study the possible mechanisms of CUR on angiogenic biomarkers, VEGF, COX-2, and EGFR.

## 2. Methods

### 2.1. Cell Line and Cell Culture

Cervical cancer cells (CaSki) were purchased from the American Type Culture Collection. The cell lines were cultured in MEM medium supplemented with 10% fetal bovine serum. All cultures were maintained in an incubator at 37°C with 5% CO_2_ in a humidified atmosphere.

### 2.2. CaSki-Induced Tumor Mice

BALB/c-nude mice weighing about 20–25 g were used. The animal experiments were conducted according to the guidelines on experimental animals of The National Research Council of Thailand (1999). The mice were divided into 6 groups: (1) controls supplemented with corn oil (Control + vehicle; *n* = 6), (2) controls supplemented with CUR (1,500 mg/kg) (Control + CUR; *n* = 6), (3) CaSki-implanted mice supplemented with corn oil (CaSki + vehicle; *n* = 6), (4) CaSki-implanted mice supplemented with CUR (500 mg/kg) (CaSki + CUR500, *n* = 6), (5) CaSki-implanted mice supplemented with CUR (1,000 mg/kg) (CaSki + CUR1000, *n* = 6), and (6) CaSki-implanted mice supplemented with CUR (1,500 mg/kg) (CaSki + CUR1500, *n* = 6).

For the CaSki groups, a suspension of 10 × 10^6^ CaSki cells in 0.2 mL MEM [14] was subcutaneously injected into the dorsa of mice at the proximal midline while control group was injected with MEM. The tumors were measured with Vernier calipers every 3-4 days by using the formula *a* ^2^ × *b* × 0.52 (where *a* is the shortest diameter and *b* is the longest diameter). When the tumor volume was 100–120 mm^3^, mice were randomized. Then, the mice were daily supplemented with vehicle or CUR (Cayman Chemical, USA) at the doses of 500, 1,000, or 1,500 mg/kg of body weight for one month.

### 2.3. Study of Tumor Microvasculature

On study day 30, the mice were anesthetized with sodium pentobarbital (50 mg/kg bw,* i.p*). Fluorescence tracers (0.1 mL of 0.5% fluorescein isothiocyanate- (FITC-) labeled dextran (MW = 200,000, Sigma Chemical, USA)) were injected in the jugular vein. The tumor microvasculature was visualized under confocal microscope.

### 2.4. Immunohistochemistry for CD31 Expression and Microvessels Density (MVD) Determination

After the microvascular study, the mice were sacrificed and the tumors were fixed in 10% formalin. Immunohistochemistry was performed using 5 *μ*m thick paraffin sections. Paraffin sections were dewaxed and rehydrated through xylene and a graded alcohol series. Endogenous peroxidase activity was blocked with 3% hydrogen peroxide for 15 min at room temperature. After washing in water, nonspecific binding sites were blocked with 5% bovine serum in phosphate-buffered saline (PBS) for 30 min at room temperature. The tissue slide samples were incubated with primary monoclonal antibody CD31 (Thermo Fischer Scientific, UK) (1 : 500) at 4°C overnight. The slide was then gently rinsed with PBS and developed by the Envision system/HRP (DAKO cytomation, USA) for 30 min and substrate-chromogen for 10 min at room temperature. The nuclei were counterstained with Mayer's hematoxylin.

To quantify angiogenesis, microvessel density (MVD) was assessed by immunostaining with the anti-CD31 antibody as previously described [15]. The sections were observed first under the low power (×40), and then the most dense area of microvessel sections was selected and counted under the high power (×200, the surface area of every vision field being 0.4 mm^2^).

### 2.5. Immunohistochemistry for VEGF, COX-2, and EGFR Expression

Paraffin sections from dorsal skin tissue were dewaxed and rehydrated with xylene and a graded alcohol series. Endogenous peroxidase activity was blocked with 3% hydrogen peroxide for 15 min at room temperature. After washing in water, nonspecific binding sites were blocked with 5% bovine serum in phosphate-buffered saline (PBS) for 30 min at room temperature. The tissue slide samples were incubated with primary monoclonal antibody VEGF (Thermo Fischer Scientific, UK) (1 : 100) or COX-2 (Thermo Fischer Scientific, UK) (1 : 50) or EGFR (VENTANA (ready to use), USA) at 4°C overnight. The slide was then gently rinsed with PBS and developed by the Envision system/HRP (DAKO cytomation, USA) for 30 min and substrate-chromogen for 10 min at room temperature. The nuclei were counterstained with Mayer's hematoxylin.

### 2.6. Staining Analysis

The sample image from each slide was subtracted from the corresponding background image. The image threshold was standardized to delineate the labeled structures and then applied to all images of an individual experiment. The staining intensity and the percentage of positive staining for VEGF, COX-2, and EGFR were analyzed. Staining intensity (*I*) was scored as 0 (none), 1+ (weak), 2+ (moderate), and 3+ (strong). The percentage of positive staining (*P*) was scored as (0) 0% immunopositive cells; (1) ≤25% positive cells; (2) 26~50% positive cells; (3) ≥51–75% positive cells; (4) ≥76%. The sum of both (*I*) and (*P*) scores was evaluated for each case and a final score was assigned 0 (negative), 1–3 (weak expression), 4-5 (moderate expression), and 6-7 (strong expression) [16].

### 2.7. Statistical Analysis

Data were expressed as means with standard error. SPSS.13 software was used for statistical analysis. Student's unpaired *t*-test was applied for comparison of the means of two groups (Control and CaSki + vehicle groups), and analysis of variance was used for the means of multiple groups. The correlation between COX-2, VEGF, and CD31 expressions and COX-2, VEGF, and EGFR was assessed with Pearson correlation test. For all of the value differences, *P* value less than 0.05 was considered significant.

## 3. Results

### 3.1. Antitumor Effect of CUR in CaSki-Implanted Mice

**Figure 1:**
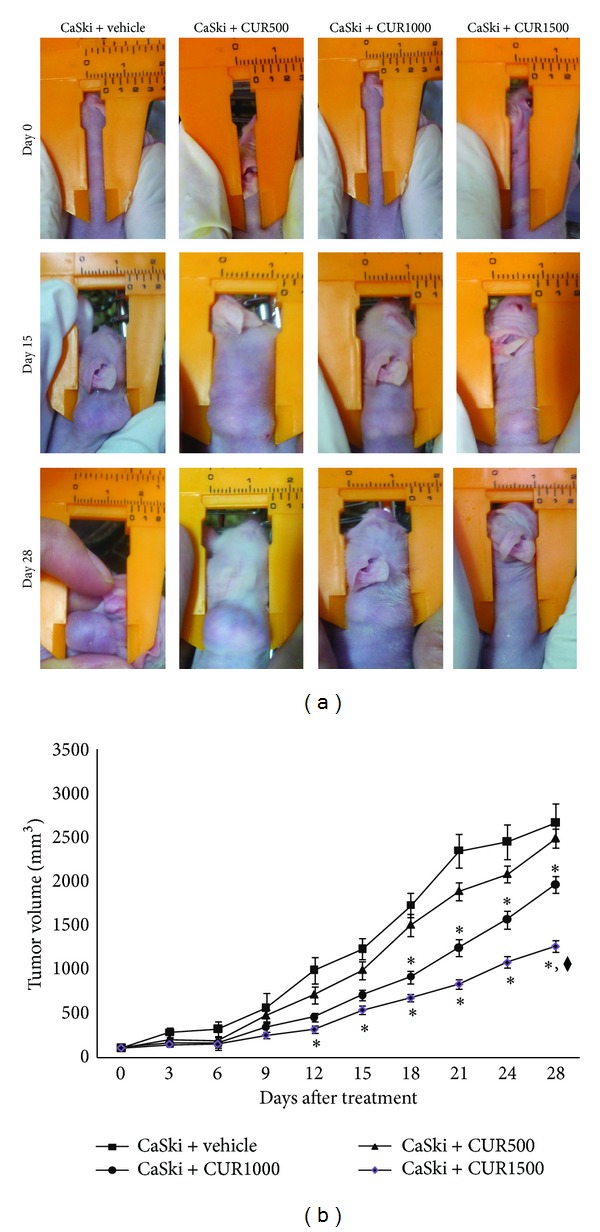
(a) Tumor bearing mice. (b) Tumor volume (mm^3^). *P < 0.001 significant difference compared to CaSki + vehicle group and CaSki + CUR500 group. ^◆^ *P* < 0.01 versus CaSki + CUR1000 group.

Tumor growth is shown in Figure 1(a). Tumors in the CaSki + vehicle group doubled in size approximately every 3 days. On day 12, the group treated with 1500 mg/kg of CUR had significantly smaller tumors (439.53 ± 23.66 mm^3^). The 1,000 mg/kg CUR group showed a significantly reduced tumor volume beginning on day 18 after treatment (918.06 ± 33.86 mm^3^). At the end of the experiment, CUR treatments at the dose of 1,000 and 1,500 mg/kg significantly retarded the growth of tumors by 21.03% and 35.57%, respectively (Figure 1(b); *P* < 0.001). Moreover, the CaSki + CUR1500 treated group (1,266.00 ± 36.41 mm^3^) showed significantly reduced tumor volume versus CaSki + CUR1000 treated group (1,964.78 ± 40.20 mm^3^) (*P* < 0.01). However, the reduction of tumor growth in CaSki + CUR500 treated group (2,487.99 ± 28.32 mm^3^) did not reach a significant level as compared with the CaSki + vehicle group (2,668.42 ± 30.36 mm^3^).

### 3.2. Antiangiogenic Effect of CUR in CaSki-Implanted Mice

**Figure 2:**
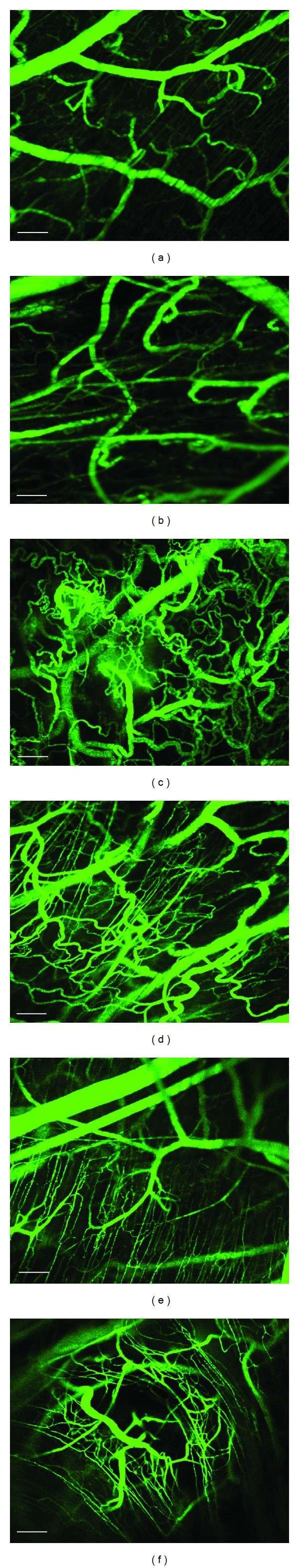
Confocal images of tumor microvasculature in Control + vehicle group (a), Control + CUR (b), CaSki + vehicle group (c), CaSki + CUR500 group (d), CaSki + CUR1000 group (e), and CaSki + CUR1500 group (f). Bar = 100 *μ*m, 10x.

The confocal fluorescent images of the microvasculature for controls (a and b), CaSki + vehicle group (c), and CaSki + CUR groups (d–f) are demonstrated in Figure 2. There was a marked increase in capillary networks in the CaSki groups. These networks were heterogeneous, tortuous, dilated, and hyperpermeable with extravasations of fluorescence tracer. However, the appearance of neocapillaries induced by CaSki was markedly reduced after treatment with high dose CUR (1,000 and 1,500 mg/kg). In addition, the abnormalities of the neocapillary network pattern were attenuated by high dose CUR.

**Figure 3:**
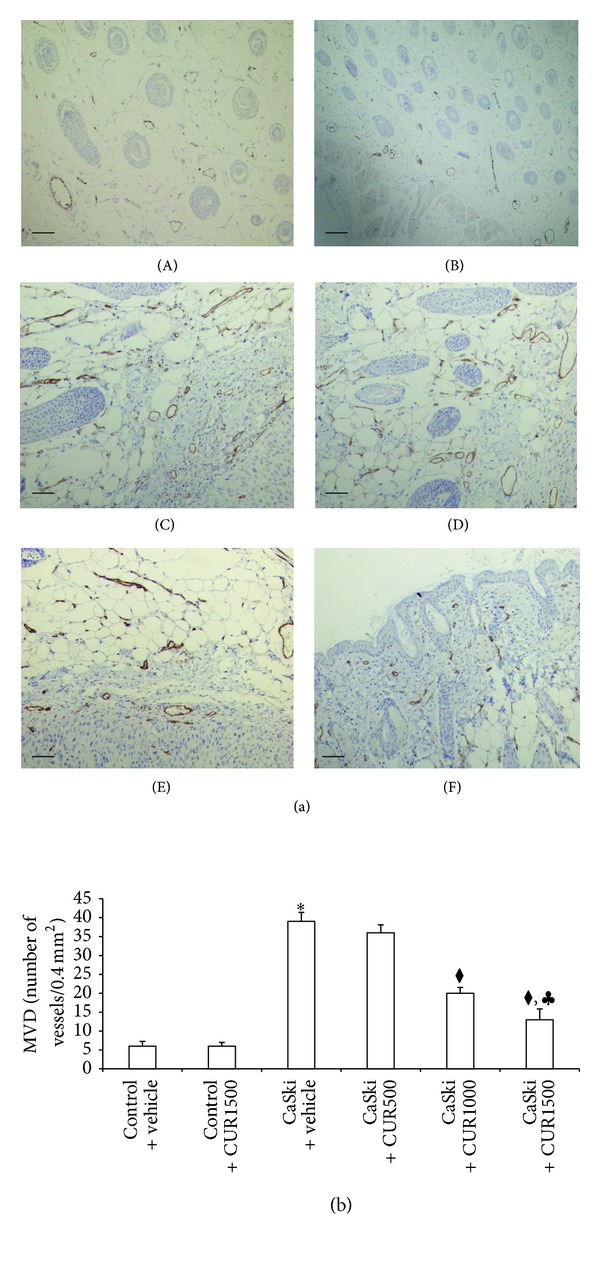
(a) Immunohistochemical staining for CD31 in Control + vehicle group (A), Control + CUR (B), CaSki + vehicle group (C), CaSki + CUR500 group (D), CaSki + CUR1000 group (E), and CaSki + CUR1500 group (F). Bar = 10 *μ*m, 200x. (b) Microvascular density (numbers/0.4 mm^2^) (mean ± SEM). *P < 0.001 versus Control + vehicle group, ^◆^ *P* < 0.001 versus CaSki + vehicle group and CaSki + CUR500 group, and ^*♣*^ *P* < 0.05 versus CaSki + CUR1,000 group.

Figure 3(a) shows representative immunostaining for CD31 in control (A and B), CaSki + vehicle (C), and CaSki + CUR (D–F) groups. In normal skin tissue from the control group, few CD31 expressions were detected adjacent to sweat glands, whereas they were highly expressed in CaSki-implanted tissues. High dose CUR attenuated CD31 expression.

In Figure 3(b), the MVD of both Control + vehicle and Control + CUR1500 was similar (6 ± 1.25 and 6 ± 1.02, resp.). The MVD was significantly higher in the CaSki + vehicle group (39 ± 2.38) than in the control group (*P* < 0.001). The MVD was not significantly decreased by CUR treatment at the dose of 500 mg/kg (36 ± 2.12). High dose CUR: 1,000 (20 ± 1.54) and 1,500 mg/kg (13 ± 2.85), was significantly decreased in MVD as compared to the CaSki + vehicle group and CaSki + CUR500 group (*P* < 0.001). Moreover, MVD in the CaSki + CUR1500 group was significantly decreased when compared to CaSki + CUR1000 group (*P* < 0.05).

### 3.3. Effects of CUR on Angiogenic Biomarkers

**Figure 4:**
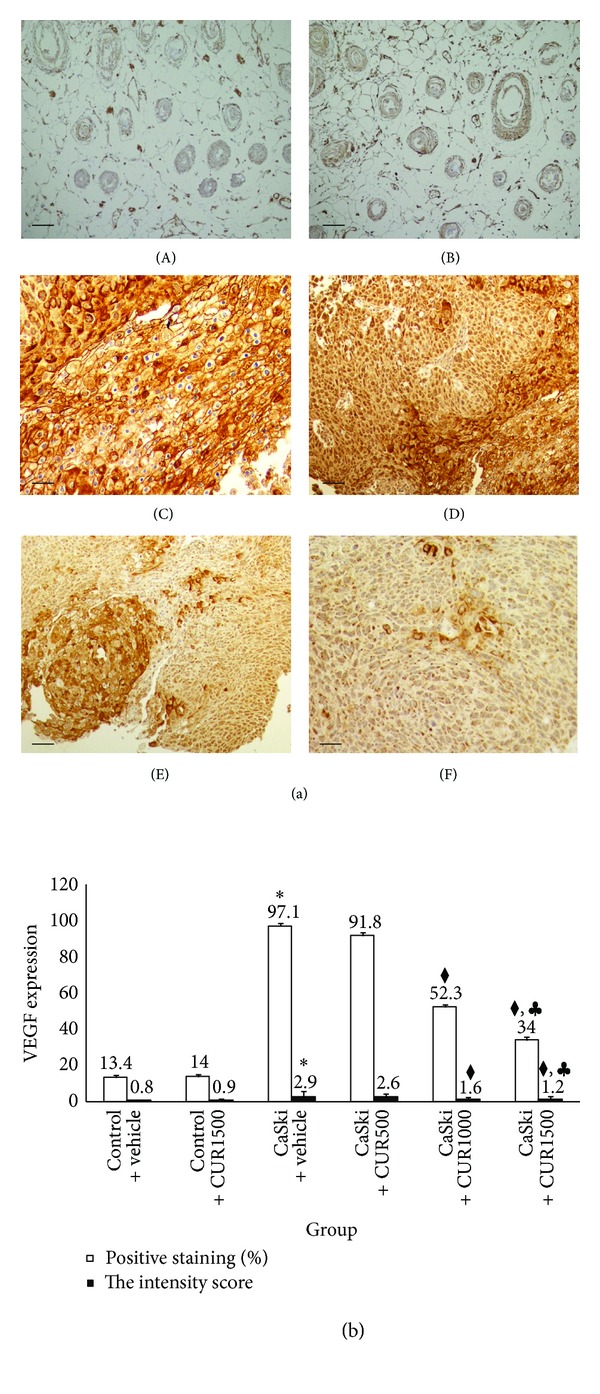
(a) Immunohistochemical staining for VEGF expression in Control + vehicle group (A), Control + CUR (B), CaSki + vehicle group (C), CaSki + CUR500 group (D), CaSki + CUR1000 group (E), and CaSki + CUR1500 group (F). Bar = 10 *μ*m, 200x. (b) The percentage of positive staining (%) and the intensity of VEGF expression (mean ± SEM). *P < 0.001 versus Control + vehicle group, ^◆^ *P* < 0.001 versus CaSki + vehicle group and CaSki + CUR500 group, and ^*♣*^ *P* < 0.001 versus CaSki + CUR1000 group.

**Figure 5:**
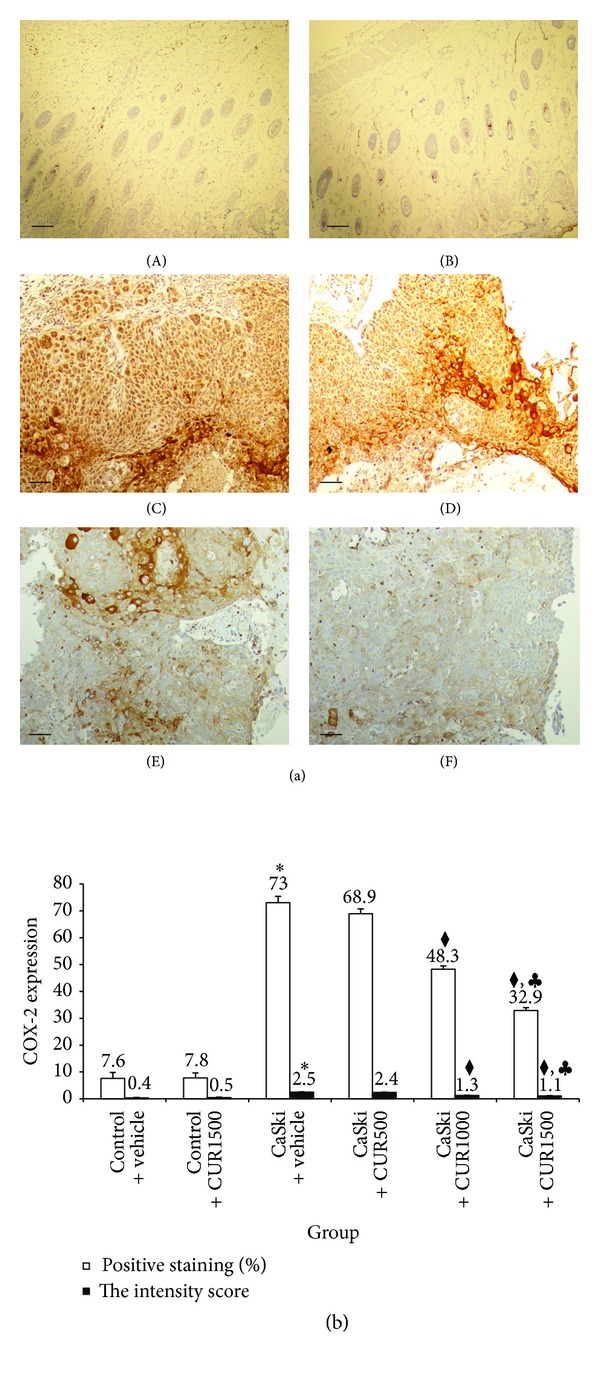
(a) Immunohistochemical staining for COX-2 expression in Control + vehicle group (A), Control + CUR (B), CaSki + vehicle group (C), CaSki + CUR500 group (D), CaSki + CUR1000 group (E), and CaSki + CUR1500 group (F). Bar = 10 *μ*m, 200x. (b) The percentage of positive staining (%) and the intensity of COX-2 expression (mean ± SEM). *P < 0.001 versus Control + vehicle group, ^◆^ *P* < 0.001 versus CaSki + vehicle group and CaSki + CUR500 group, and ^*♣*^ *P* < 0.001 versus CaSki + CUR1000 group.

Figures 4(a) and 5(a) show the microscopic images of immunohistochemical stained sections for VEGF and COX-2 expression, respectively. Cytoplasm of the tumor cells stained positively for VEGF and COX-2. Figures 4(b) and 5(b) show the percentage of positive staining of VEGF and COX-2, respectively. The percentage of positive staining of VEGF (97.10 ± 2.17%) and COX-2 (73.00 ± 1.23%) expression was significantly increased in the CaSki + vehicle group as compared to control group (VEGF: 13.40 ± 1.17%; COX-2: 7.60 ± 1.27%) (*P* < 0.001).

The percentage of positive staining of VEGF, which was 91.80 ± 3.32%, 52.30 ± 3.20%, and 34.00 ± 1.93%, respectively, was attenuated by the treatments with CUR at the doses of 500, 1,000, and 1,500 mg/kg but was only significantly different in the high dose groups (*P* < 0.001). The treatment with CUR at the dose of 1,500 mg/kg has also shown a more significant decrease in VEGF positive staining than the treatment with CUR at the dose of 1,000 mg/kg (*P* < 0.001).

The percentage of positive staining of COX-2, which was 68.90 ± 1.83% and 48.30 ± 1.21%, and 32.90 ± 1.08% at the doses of 500, 1,000, and 1,500 mg/kg, respectively, was attenuated by the treatments with CUR. In the same fashion, a significant reduction in COX-2 positive staining was found only in the two high dose groups (*P* < 0.001). The treatment with CUR at the dose of 1,500 mg/kg showed a significant decrease in COX-2 positive staining versus the 1,000 mg/kg dose (*P* < 0.001).

The intensity scores of VEGF and COX-2 expression are shown in Figures 4(b) and 5(b). The CaSki + vehicle group showed strong intensity scores for VEGF (mean score = 2.9) and COX-2 (mean score = 2.5), whereas the control group showed weak intensity scores (mean score = 0.8 and 0.4 for VEGF and COX-2, resp.). Staining intensity scores for VEGF expression were reduced in CaSki + CUR500 group (mean score = 2.6), CaSki + CUR1000 group (mean score = 1.6), and CaSki + CUR1500 group (mean score = 1.2) but were significantly different only in the two high dose groups (*P* < 0.001).

In the same fashion, staining intensity scores for COX-2 expression were reduced in the CaSki + CUR500 group (mean score = 2.4), CaSki + CUR1000 group (mean score = 1.3), and the CaSki + CUR1500 group (mean score = 1.1) but were significantly different only in the two high dose groups (*P* < 0.001).

The total scores for VEGF positive staining and intensity revealed that the CaSki + vehicle group had strong VEGF expression (mean total score = 6.9), whereas the control group had weak VEGF expression (mean total score = 1.8). The CaSki + vehicle group had moderate COX-2 expression (mean total score = 5.6), whereas the control group had weak COX-2 expression (mean total score = 1.4).

In the treated groups, the total scores for VEGF expression revealed strong, moderate, and weak expressions in CaSki + CUR500 group (mean total score = 6.6), CaSki + CUR1,000 group (mean total score = 4.1), and CaSki + CUR1500 group (mean total score = 3.3), respectively. The total scores for COX-2 expression showed that moderate, weak, and weak expressions were found in CaSki + CUR500 group (mean total score = 5.4), CaSki + CUR1000 group (mean total score = 3.5), and CaSki + CUR1500 group (mean total score = 3.1), respectively.

### 3.4. Effects of CUR on EGFR

**Figure 6:**
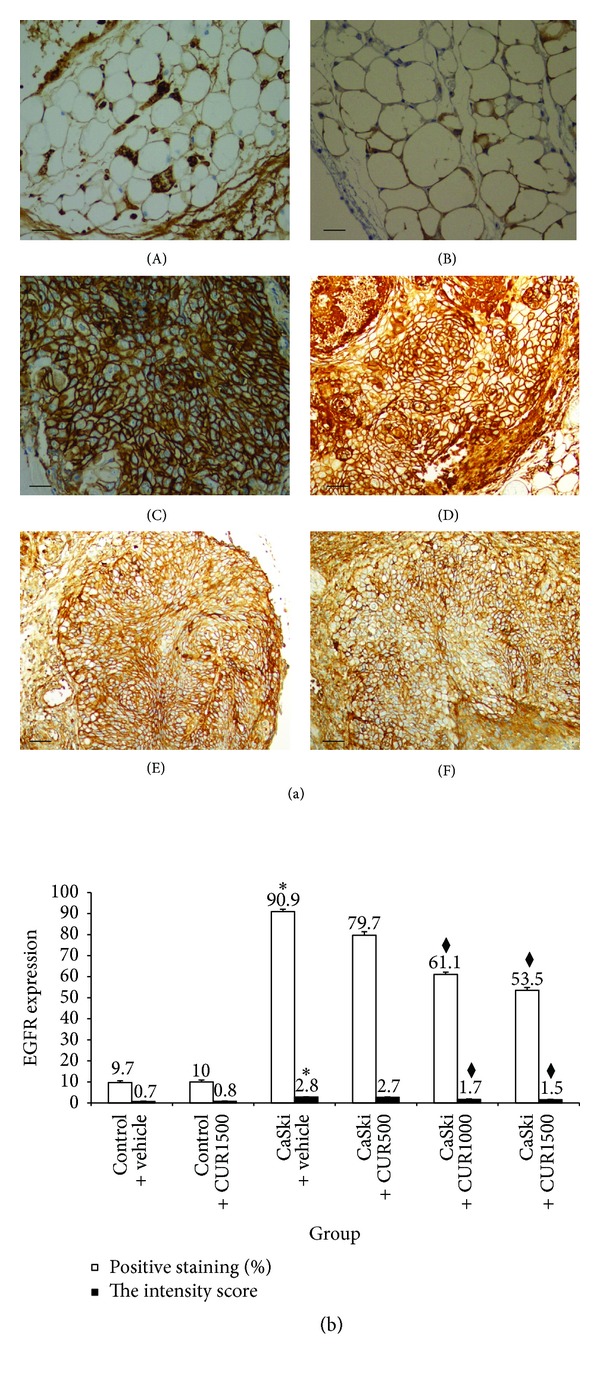
(a) Immunohistochemical staining for EGFR expression in Control + vehicle group (A), Control + CUR (B), CaSki + vehicle group (C), CaSki + CUR500 group (D), CaSki + CUR1000 group (E), and CaSki + CUR1500 group (F). Bar = 10 *μ*m, 200x. (b) The percentage of positive staining (%) and the intensity of EGFR expression (mean ± SEM). *P < 0.001 versus Control + vehicle group and ^◆^ *P* < 0.001 versus CaSki + vehicle group and CaSki + CUR500 group.

The EGFR staining pattern was predominantly in the membrane with occasional cytoplasmic positivity (Figure 6(a)). The quantitative data showed that the percentage of positive staining of EGFR expression significantly increased in the CaSki + vehicle group (90.90 ± 1.26%) compared to control group [9.70 ± 0.79%, *P* < 0.001 (Figure 6(b))]. The percentage of positive staining of EGFR, which was 79.70 ± 1.26%, 61.10 ± 1.03%, and 53.50 ± 1.31%, respectively, was attenuated by the treatments with CUR at the doses of 500, 1,000, and 1,500 mg/kg. Significant reductions in EGFR positive staining were found only in high dose groups (*P* < 0.001).

The intensity score of EGFR expression in CaSki + vehicle group was high (mean score = 2.8) but was low (mean score = 0.7) in the control group (Figure 6(b)). However, staining intensities for EGFR expression were reduced in CaSki + CUR500 (mean score = 2.7), CaSki + CUR1000 (mean score = 1.7), and CaSki + CUR1500 (mean score = 1.5) groups. Again, significance was only achieved in the two high dose groups (*P* < 0.001).

The total score for EGFR expression showed that the CaSki + vehicle group had strong EGFR expression (mean total score = 6.8), whereas the control group had weak EGFR expression (mean total score = 1.7). The EGFR expression extent and intensity scores in the treated group revealed that strong, moderate, and moderate expressions were found in CaSki + CUR500 (mean total score = 6.5), CaSki + CUR1000 (mean total score = 4.7), and CaSki + CUR1500 groups (mean total score = 4.1), respectively.

### 3.5. The Relationship between the Expression of CD31, VEGF, COX-2, and EGFR

The percentage of stained positive cells for both VEGF and COX-2 expressions was positively correlated and related to MVD in CaSki-implanted mice (*r* = 0.99 and *r* = 0.95, resp., *P* < 0.001) as were the respective intensity scores (*r* = 0.877 and *r* = 0.851, *P* < 0.001). Furthermore, VEGF expression and intensity scores were positively correlated with both COX-2 and EGFR expressions: (i) *r* = 0.921 and *r* = 0.964, respectively, (*P* < 0.001) and (ii) *r* = 0.821 and *r* = 0.866, respectively, (*P* < 0.001).

## 4. Discussion

In the present study, the experiments were conducted to investigate the effects of CUR on tumor progression and angiogenesis using CaSki-implanted nude mice model. We demonstrated that CUR exhibits antitumor and antiangiogenesis effects in CaSki-implanted nude mice.

One of the mechanisms by which CUR attenuates tumor progression is mediated by its antiangiogenic activity. In the original hypothesis formulated by Folkman in the early 1970s of angiogenic control of tumor growth [17], it was proposed that tumor growth was limited by diffusion to a size of 1-2 mm unless additional blood vessels were recruited to the tumor site. Moreover, it is important to realize that the rate of cell proliferation in such tumors is virtually the same as in rapidly expanding tumors. This has been shown by the work of Hanahan and Folkman, who have followed tumor development in transgenic mice that develop pancreatic islet tumors. They demonstrated that the initial phase of tumor growth is characterized by avascular tumors that maintain a small diameter for a period of weeks until an angiogenic switch is activated and the tumors become vascular and begin to expand in size [18]. Treatment of such mice with angiogenesis inhibitors blocked formation of these tumor colonies [19]. These results indicate that angiogenesis inhibition can lead to tumor regression and, in some cases, to complete elimination of the tumor growth.

In normal tissues, angiogenesis is strictly controlled but in tumors angiogenesis is uncontrolled and immature [20]. Controlled by angiogenic factors and angiogenic inhibitors, tumor cells, endothelial cells, and other cells can produce and release VEGF protein if the local microenvironment is changed by hypoxia [21]. In cervical cancer, human papillomavirus may directly stimulate VEGF production through the upregulation of the E6 oncoprotein [22, 23]. Studies using transgenic mice as well as samples of human cervical tissue suggest that, apart from hypoxia, E6 and E7 oncoproteins can also stimulate VEGF production [24]. López-Ocejo et al. have demonstrated that E6 positive cervical carcinoma cells expressed VEGF mRNA levels two to three times higher than those expressed by E6 negative cells [23]. On the other hand, expression of the antiangiogenic factors thrombospondin- (TSP-) 1 and (TSP-) 2 was decreased in cells infected by HPV. Consequently, it would appear that cervical cancer expression of HPV-16 (CaSKi cell) integrated molecules is able to contribute to a proangiogenic phenotype that might support tumor growth and angiogenesis via upregulation of VEGF expression.

The VEGF pathway plays a crucial role in normal and pathologic angiogenesis, triggering multiple signaling networks that result in endothelial cell survival, migration, proliferation, differentiation, and vascular permeability [25]. In our study, we found that there was a marked increase in neovascularization with a heterogeneous network, hyperpermeability to macromolecules, tortuosity, and dilatation in the CaSki groups as compared to the control group. Moreover, we demonstrated that a strong correlation was found between VEGF expression and increased tumor microvasculature in CaSki + vehicle group. These results suggest strongly that VEGF and angiogenesis promoted by VEGF play important roles in tumor growth. We also found clear heterogeneity in VEGF expression and new vessel formation in cancer tissue. The VEGF expression and MVD were highly correlated in the cancer tissues again suggesting strongly the link between VGF and MVD and the important roles in tumor biological behavior and progression.

Similarly, COX-2 overexpression in CaSki + vehicle group was highly and positively correlated with MVD, providing strong support for its role in tumor-induced angiogenesis in cervical cancer. COX-2 can potentially predispose to cervical cancer by several direct and indirect mechanisms, for example, inhibiting apoptosis, suppressing immune function, promoting angiogenesis, and enhancing the invasiveness of malignant cells [8]. The direct effect of COX-2 on angiogenesis has been demonstrated by Dormond et al. [26]. They showed that inhibition of endothelial cell COX-2 by nonsteroidal anti-inflammatory drugs suppressed *α*V*β*3-dependent activation of the small GTPases, Cdc-42, and Rac, resulting in inhibition of endothelial cell spreading and migration* in vitro* [26]. Therefore, COX enzymes appear essential for the maintenance of the migration and attachment of endothelial cells through integrin pathways. The indirect pathway of COX-2 on tumor angiogenesis might be mediated by an upregulation of the expression of angiogenic factors like VEGF. This pathway of COX-2 in angiogenesis is thought to be the induction of the synthesis of prostanoids, which then stimulate the expression of proangiogenic factors [10]. Taken together, these findings suggest that both COX-2 and VEGF appear crucial for tumor angiogenesis in cervical cancer, and therapy targeting COX-2 and VEGF pathways should be explored.

Furthermore, we demonstrated that strong expression of EGFR was found in CaSki-implanted mice and that COX-2, VEGR, and EGFR were strongly positively related. We hypothesize that overexpression of the angiogenic biomarkers COX-2 and VEGF may be mediated by the induction of EGFR signaling pathway. EGFR has recently been identified as a promising target for cervical cancer [27]. This receptor is overexpressed in a variety of solid human cancers, such as non-small-cell lung cancer, colorectal cancer, and head and neck cancer. In patients with squamous cell carcinoma of cervix, EGFR is overexpressed in up to 85% of cases, and EGFR expression has been associated with a later tumor stage and a poorer prognosis [13, 28, 29].

Kulkarni et al. found that EGF and TGF-*α* ligands of EGFR markedly induced COX-2 in a cervical carcinoma cell line [9]. This suggests that deregulated signaling through EGFR is likely to account, at least in part, for increased expression of COX-2 in cervical cancer. Later, Oh et al. reported that COX-2-prostaglandin E2 (PGE2) pathway is also implicated in VEGF expression by HPV 16 E5 [10]. Collectively, these data provide evidence for an association between EGFR, COX-2, and VEGF expressions in cervical cancer tumor angiogenesis and tumor growth.

In our study, we have demonstrated that the CUR treatment at the doses of 1,000 and 1,500 mg/kg inhibited tumor growth and angiogenesis and that such tumor-associated pathological features as microvascular dilatation, tortuosity, and hyperpermeability were attenuated. Consistent with data from others [28, 29] and our previous work [27], CUR inhibited the expressions of VEGF, COX-2, and EGFR. Interestingly, these high doses did not result in any deaths or overt toxicity.

## 5. Conclusion

Our data demonstrated that CUR markedly inhibited tumor progression and angiogenesis in CaSki-implanted nude male mice models. The antiangiogenic effects of CUR partially through VEGF and COX-2 suppression might be mediated by downregulation of EGFR. Our data suggest a potential clinical role for the treatment of cervical cancer and should be explored further.

## References

[1] Cuzick J, Arbyn M, Sankaranarayanan R (2008). Overview of human papillomavirus-based and other novel options for cervical cancer screening in developed and developing countries. *Vaccine*.

[2] Rogers SJ, Harrington KJ, Rhys-Evans P, O-Charoenrat P, Eccles SA (2005). Biological significance of c-erbB family oncogenes in head and neck cancer. *Cancer and Metastasis Reviews*.

[3] Kim S-H, Juhnn Y-S, Kang S (2006). Human papillomavirus 16 E5 up-regulates the expression of vascular endothelial growth factor through the activation of epidermal growth factor receptor, MEK/ERK1,2 and PI3K/Akt. *Cellular and Molecular Life Sciences*.

[4] Balan R, Simion N, Giusca SE (2011). Immunohistochemical assessment of p16, COX-2 and EGFR in HPV-positive cervical squamous intraepithelial lesions. *Romanian Journal of Morphology and Embryology*.

[5] Kerbel RS (2008). Tumor angiogenesis. *The New England Journal of Medicine*.

[6] Cheng WF, Chen CA, Lee CN, Wei LH, Hsieh F, Hsieh C (2000). Vascular endothelial growth factor and prognosis of cervical carcinoma. *Obstetrics and Gynecology*.

[7] Kim MH, Seo SS, Song YS (2003). Expression of cyclooxygenase-1 and -2 associated with expression of VEGF in primary cervical cancer and at metastatic lymph nodes. *Gynecologic Oncology*.

[8] Chun KS, Surh YJ (2004). Signal transduction pathways regulating cyclooxygenase-2 expression: potential molecular targets for chemoprevention. *Biochemical Pharmacology*.

[9] Kulkarni S, Rader JS, Zhang F (2001). Cyclooxygenase-2 is overexpressed in human cervical cancer. *Clinical Cancer Research*.

[10] Oh JM, Kim SH, Lee YI (2009). Human papillomavirus E5 protein induces expression of the EP4 subtype of prostaglandin E2 receptor in cyclic AMP response element-dependent pathways in cervical cancer cells. *Carcinogenesis*.

[11] Singh M, Singh N (2009). Molecular mechanism of curcumin induced cytotoxicity in human cervical carcinoma cells. *Molecular and Cellular Biochemistry*.

[12] Downs LS, Rogers LM, Yokoyama Y, Ramakrishnan S (2005). Thalidomide and angiostatin inhibit tumor growth in a murine xenograft model of human cervical cancer. *Gynecologic Oncology*.

[13] Yoysungnoen P, Wirachwong P, Changtam C, Suksamram A, Patumraj S (2008). Anti-cancer and anti-angiogenic effects of curcumin and tetrahydrocurcumin on implanted hepatocellular carcinoma in nude mice. *World Journal of Gastroenterology*.

[14] Mahasiripanth T, Hokputsa S, Niruthisard S, Bhattarakosol P, Patumraj S (2012). Effects of Acanthus ebracteatus Vahl on tumor angiogenesis and on tumor growth in nude mice implanted with cervical cancer. *Journal of Cancer Management and Research*.

[15] Weidner N, Semple JP, Welch WR, Folkman J (1991). Tumor angiogenesis and metastasis—correlation in invasive breast carcinoma. *New England Journal of Medicine*.

[16] Kolev Y, Uetake H, Iida S, Ishikawa T, Kawano T, Sugihara K (2007). Prognostic significance of VEGF expression in correlation with COX-2, microvessel density, and clinicopathological characteristics in human gastric carcinoma. *Annals of Surgical Oncology*.

[17] Folkman J (1971). Tumor angiogenesis: therapeutic implications. *New England Journal of Medicine*.

[18] Hanahan D, Folkman J (1996). Patterns and emerging mechanisms of the angiogenic switch during tumorigenesis. *Cell*.

[19] Parangi S, O’Reilly M, Christofori G (1996). Antiangiogenic therapy of transgenic mice impairs de novo tumor growth. *Proceedings of the National Academy of Sciences of the United States of America*.

[20] Saaristo A, Karpanen T, Alitalo K (2000). Mechanisms of angiogenesis and their use in the inhibition of tumor growth and metastasis. *Oncogene*.

[21] Goldberg MA, Schneider TJ (1994). Similarities between the oxygen-sensing mechanisms regulating the expression of vascular endothelial growth factor and erythropoietin. *Journal of Biological Chemistry*.

[22] Toussaint-Smith E, Donner DB, Roman A (2004). Expression of human papillomavirus type 16 E6 and E7 oncoproteins in primary foreskin keratinocytes is sufficient to alter the expression of angiogenic factors. *Oncogene*.

[23] López-Ocejo O, Viloria-Petit A, Bequet-Romero M, Mukhopadhyay D, Rak J, Kerbel RS (2000). Oncogenes and tumor angiogenesis: the HPV-16 E6 oncoprotein activates the vascular endothelial growth factor (VEGF) gene promoter in a p53 independent manner. *Oncogene*.

[24] Coussens LM, Hanahan D, Arbeit JM (1996). Genetic predisposition and parameters of malignant progression in K14- HPV16 transgenic mice. *American Journal of Pathology*.

[25] Pan MH, Huang TM, Lin JK (1999). Biotransformation of curcumin through reduction and glucuronidation in mice. *Drug Metabolism and Disposition*.

[26] Dormond O, Foletti A, Paroz C, Rüegg C (2001). NSAIDS inhibit *α* V *β* 3 integrin-mediated and Cdc42/Rac-dependent endothelial-cell spreading, migration and angiogenesis. *Nature Medicine*.

[27] Yoysungnoen P, Wirachwong P, Bhattarakosol P, Niimi H, Patumraj S (2005). Antiangiogenic activity of curcumin in hepatocellular carcinoma cells implanted nude mice. *Clinical Hemorheology and Microcirculation*.

[28] Aggarwal S, Takada Y, Singh S, Myers JN, Aggarwal BB (2004). Inhibition of growth and survival of human head and neck squamous cell carcinoma cells by curcumin via modulation of nuclear factor-*κ*B signaling. *International Journal of Cancer*.

[29] Sandur SK, Ichikawa H, Pandey MK (2007). Role of pro-oxidants and antioxidants in the anti-inflammatory and apoptotic effects of curcumin (diferuloylmethane). *Free Radical Biology and Medicine*.

